# Aggressive and psychopathic traits are linked to the acquisition of stable but imprecise hostile expectations

**DOI:** 10.1038/s41398-023-02497-0

**Published:** 2023-06-10

**Authors:** Macià Buades-Rotger, Danique Smeijers, David Gallardo-Pujol, Ulrike M. Krämer, Inti A. Brazil

**Affiliations:** 1 Radboud University, Donders Institute for Brain, Cognition and Behaviour, Nijmegen, The Netherlands; 2 Department of Neurology, University of Lübeck, Lübeck, Germany; 3 Department of Clinical Psychology and Psychobiology, University of Barcelona, Barcelona, Spain; 4 Division Diagnostics, Research, and Education, Forensic Psychiatric Center Pompestichting, Nijmegen, The Netherlands; 5 Behavioural Science Institute, Radboud University, Nijmegen, The Netherlands; 6 Institute of Neurosciences, Barcelona, Spain; 7 Department of Psychology, University of Lübeck, Lübeck, Germany; 8 Center of Brain, Behavior and Metabolism (CBBM), University of Lübeck, Lübeck, Germany

**Keywords:** Human behaviour, Long-term memory

## Abstract

Individuals with hostile expectations (HEX) anticipate harm from seemingly neutral or ambiguous stimuli. However, it is unclear how HEX are acquired, and whether specific components of HEX learning can predict antisocial thought, conduct, and personality. In an online sample of healthy young individuals (*n* = 256, 69% women), we administered a virtual shooting task and applied computational modelling of behaviour to investigate HEX learning and its constellation of correlates. HEX acquisition was best explained by a hierarchical reinforcement learning mechanism. Crucially, we found that individuals with relatively higher self-reported aggressiveness and psychopathy developed stronger and less accurate hostile beliefs as well as larger prediction errors. Moreover, aggressive and psychopathic traits were associated with more temporally stable hostility representations. Our study thus shows that aggressiveness and psychopathy are linked with the acquisition of robust yet imprecise hostile beliefs through reinforcement learning.

## Introduction

Forming accurate representations (i.e., beliefs) concerning others is crucial to navigate the social world [1–3]. The accuracy of these beliefs may, however, be distorted in the presence of certain psychopathological traits [4, 5], possibly contributing to the development of maladaptive and disruptive social behaviour. In particular, antisocial individuals tend to expect hostile outcomes in seemingly neutral or ambiguous social encounters [6–10]. Such Hostile Expectations (HEX) belong to a set of interrelated hostility biases found across aggressive populations [11]. Yet, it is still unclear how HEX are acquired, and whether the propensity to develop HEX relates to various established correlates of antisocial behaviour, such as aggressiveness, psychopathic traits, risk-taking, or reward and punishment sensitivity [12]. In the present study, we set to characterize HEX learning and its concomitants in depth, in line with the notion that effectively managing antisocial behaviour requires a thorough understanding of its underlying mechanisms [13, 14]. Failure to do so comes at a cost: the global economic expenditure due to interpersonal violence has been estimated to be as high $14.76 trillion in 2017, which corresponds to 12.4% of the world’s gross domestic product, or $1988 per person [15].

Here, we propose that the tendency to form HEX reflects a reinforcement learning disruption in which non-threatening events are erroneously associated with hostile outcomes. This notion is well-aligned with a growing body of research showing reinforcement learning deficits among antisocial individuals [16–19]. Recent studies have shown that reinforcement learning in social contexts is partly driven by individuals’ beliefs concerning the volatility of the environment [3, 20], the belief that a given outcome will follow [21], the accuracy of this belief [4, 5], and the discrepancy between actual and expected outcomes, i.e., the prediction error [22, 23]. From this perspective, alterations in any of these processes could be linked to the acquisition of HEX. In the present study, we employed a computational approach to investigate HEX acquisition in a task that, unlike previous studies, required the generation of explicit hostile or non-hostile responses during an interpersonal conflict. Furthermore, we tested whether HEX acquisition varied in a high- relative to a low-threat context, as aversive environments may encourage the development of a hostile mindset [10, 24].

Crucially, we also addressed whether HEX learning was linked to other domains of hostile and antisocial behaviour, a fundamental step towards bridging computational quantities and real-world observations. To this end, associations between the computational parameters capturing HEX learning, psychopathic personality traits and multiple correlates of aggression were investigated. Two structural equation models were built to test these relationships. The first model investigated which HEX learning parameters (i.e., belief volatility, strength and accuracy of the belief, prediction error, and exploration readiness) were linked with self-report measures of physical and verbal aggression, anger, and hostility. The second model tested whether the same learning parameters could predict behaviours indexing the interpersonal, affective, lifestyle-related, and antisocial facets of psychopathy.

Previous work suggested that decreased belief volatility is associated with paranoid ideation [21, 25], state anxiety [26], *better* emotional coping [27], and psychosis [28] (but see [29]), and that belief accuracy covaries negatively with psychopathic traits [4]. Furthermore, hallucination-prone individuals generate stronger beliefs in progressively weaker stimulus-outcome associations [28]. Building upon these findings, we expected that heightened and imprecise, but more stable hostility beliefs would be linked with greater levels of psychopathy and aggressiveness, as paranoia and suspiciousness are established risk factors for aggressive behaviour [30]. In addition, we expected that increments in these variables would correlate with larger prediction errors, given that antisocial persons show a greater sensitivity to HEX violations [31, 32]. Importantly, we further tested whether HEX learning was associated with hostile appraisals of human faces [33, 34] and ambiguous social situations [35], as antisocial individuals often perceive these stimuli as threatening and this might play a role in triggering acts of aggression [11]. Finally, we investigated whether HEX acquisition was associated with risk-taking [36] as well as sensitivity to reward and punishment [37], because people with antisocial tendencies are often impulsive and risk-prone [38]. We thus aimed to comprehensively delineate the constellation of constructs associated with HEX acquisition, ranging from hostile perceptual biases to overt antisocial behaviour. While previous investigations have relied on standard Pavlovian [4, 28] or instrumental learning paradigms [20, 26], the task used here required overt aggressive vs. non-aggressive responses and thus more directly tapped into participants’ hostile tendencies. Furthermore, by punishing errors in some blocks of trials but not in others, we were able to inspect how HEX emerge under more relative to less threatening contexts.

## Methods

### Participants

In this section, we report all data exclusions, experimental manipulations, and measures in the study as well as the achieved statistical power. Participants were recruited via the electronic Radboud research participation system, which is mostly composed of students and former university students. A total of 256 participants provided complete questionnaire responses (age = 23.39 ± 7.23 [mean ± standard deviation], 69% women, 88% right-handed). Most participants were Dutch (68%) or German (16%), with no more than 3% holding any other specific nationality. A majority of participants had completed at least secondary education (58% high school diploma, 21% bachelor’s degree, 17% master’s degree, 1% did not say), few smoked tobacco (96% non-smokers), most did not consume cannabis products often (64% never, 8% less than once year, 16% once or twice a year, 6% once a month, 3% once a week, <1% daily), and predominantly drank alcohol in moderation (11% never, 1% less than once year, 13% once or twice a year, 32% once a month, 39% once a week, 1% daily).

After excluding participants with >25% missing trials, data from *n* = 269 and *n* = 251 participants were available for the Hostile Expectation Learning Task and the Hostile Interpretation Bias Task, respectively (see Results for further sensitivity analyses). Twenty-six participants with computational parameter values above or below two standard deviations relative to the sample mean were excluded because their estimates became unrealistically extreme in the data-fitting process. Such deviance might be driven by these participants’ low shoot percentage, particularly in low threat blocks (see Fig. S1). Excluded participants did not statistically differ from the rest in any demographic or self-reported variables (see also Fig. S1). Analyses relating different measures are based on subsamples (minimum *n* = 188) due to participants failing to correctly complete one or more parts of the study. Using two-tailed tests at *p* < 0.05, a sample size of 188 affords 78% power to detect correlations of r = 0.2 and 98% power to correlations of r = 0.3, which can be respectively considered medium and large true effects according to recent benchmarks for individual differences research [39]. The study was approved by the Ethics Committee of the Faculty of Social Sciences, Radboud University (code ECSW-2020-092) and all participants provided informed consent.

### Hostile expectation (HEX) learning task

**Fig. 1:**
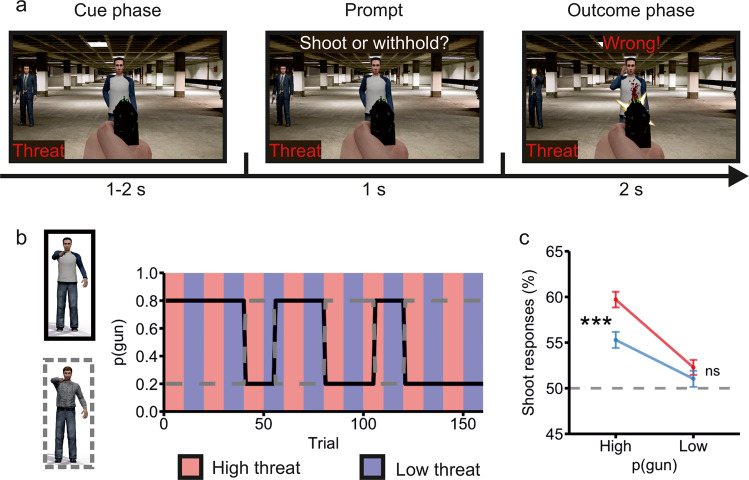
Outline of the Hostile Expectation (HEX) learning task and behavioural results. **a** Hostile Expectation (HEX) learning task. In the cue phase, participants were first presented with one of two opponents as well as a policeman in the background, with the condition (high or low threat) indicated by the word “threat” or “safe” in the lower left corner. Then came a prompt, upon which participants had one second to either shoot or withhold their gun depending on whether they predicted the man to draw a gun or a phone. In the outcome phase they obtained feedback on their decision. Wrong or missing responses led to the participant being shot by the man (if he drew a gun) or the policeman in the background (if the man drew a phone). In high threat trials, wrong decisions were punished by a loss of points, which allegedly corresponded to an undisclosed amount of money. In low threat trials, no points or money were at stake. High and low threat trials were otherwise identical. **b** Probability schedule of the task. Gun probability, termed *p*(gun), was flipped between the two opponents throughout the task. **c** Main behavioural results in the HEX learning task. Participants shot more frequently when gun probability was high and under high threat. Values are mean ± standard error. The dashed lined represents chance level. ***p < 0.001 and BF (Bayes Factor)>100. ns not significant.

To measure HEX learning, we incorporated an associative learning component into a well-validated Go-NoGo shooting task [40, 41] (Fig. 1a and b). Participants first saw a man with his hands behind his back and a policeman in the background (cue phase, 1–2 s). Then, when a prompt appeared, participants had to either shoot or withdraw their weapon depending on whether they expected the man in front of them to draw a gun or a phone (prompt, 1 s). Finally, they received feedback on their decision (outcome phase, 2 s). Wrong or missing responses resulted in the participant being shot either by the man (if he drew a gun) or the policeman in the background (if the man drew a phone). The probability of each man drawing a gun was either 0.8 or 0.2 and was flipped throughout the task for a total of 160 trials. Probabilities switched after blocks of 40, 15, 25, 25, 15, and 40 trials. Environmental threat was manipulated by including high and low threat trial blocks. In high threat blocks, participants could lose points if they chose the wrong option, whereas in low threat blocks they could not lose any points. These points allegedly corresponded to an undisclosed sum to be deducted from participants’ endowment, but in reality, they all received the same amount and were debriefed regarding the true nature of the task after completing the study. High and low threat trials were identical in all other respects. These blocks were regular, predictable, and orthogonal to gun probabilities, and were indicated in the lower left corner (Figs. 1a and b). Afterwards, participants completed the Hostile Interpretation Bias Task along with a battery of self-reports in English language.

### Hostile interpretation bias task (HIBT)

**Fig. 2:**
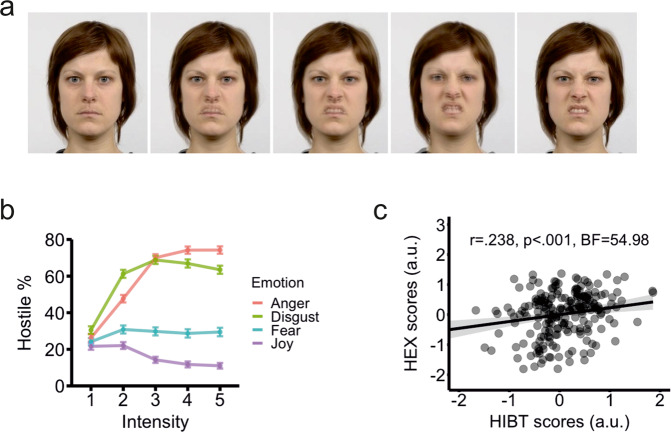
Outline of the Hostile Interpretation Bias Task (HIBT) and behavioural results. **a** Face stimuli used in the Hostile Interpretation Bias Task (HIBT). Angry, disgusted, fearful, and happy expressions were overlaid on the same actor’s neutral face to generate progressively more intense emotions. **b** Percentage of faces interpreted as hostile as a function of emotion and intensity. Values are mean ± standard error. **c** Hostile appraisals of angry faces were correlated with shoot decisions under high threat in the Hostile Expectation (HEX) learning task. r: Pearson correlation coefficient, *p*: *p*-value, BF Bayes Factor for the alternative hypothesis.

In this task, participants must indicate whether they perceived a face as hostile or not. Both options were displayed in the lower corners of the screen and participants had up to 4 s to respond. Faces varied in the emotion they depicted (angry, happy, disgusted, or fearful) and in the intensity (20%, 40%, 60%, 80%, and 100%) of the expression [34]. The intensity manipulation was crafted by overlaying an actor’s emotional expression over that same actor’s neutral expression at varying levels of transparency (Fig. 2a). Each combination of expression and intensity was presented eight times, in addition to eight neutral trials for a total of 168 plus 16 practice trials. Hostile appraisals in this task correlate with self-reported aggression [34].

### Questionnaires

The 29-item Buss-Perry Aggression Questionnaire (BPAQ) measures four facets of aggressiveness: physical aggression, verbal aggression, anger and hostility [42]. Participants had to indicate how characteristic of them each statement was (e.g., “I get into fights a little more than the average person”) on a 1 to 5 scale. Reliability was excellent in the present sample (Cronbach’s alpha = 0.90, McDonald’s omega = 0.93) and scores were in line with Dutch norms [43].

The Self-Report Psychopathy Scale-Short Form (SRP-SF) has 29 items divided in four scales [44]: interpersonal manipulation (e.g., pathological lying), callous affect (e.g., low empathy, lack of remorse), erratic lifestyle (e.g., recklessness and impulsivity), and criminal tendencies (e.g., overt antisociality, criminal activity). Participants had to indicate their agreement with each statement (e.g., “You should take advantage of other people before they do it to you”) in a 1 to 5 scale. We excluded one item inquiring on gang-related activity, following recommendations for community samples [44]. The SRP-SF displayed excellent reliability in our sample (Cronbach’s alpha = 0.90, McDonald’s omega = 0.93). Scores were normative for Dutch speakers [44].

The Word Sentence Association Paradigm – Hostility (WSAP-H) consists of sixteen short descriptions of ambiguous situations (e.g., “Someone bumps into you”) presented twice, once followed by a hostile (e.g., “rude”) and once followed by a benign (e.g., “distracted”) adjective [35]. Participants had to indicate how related they deemed the situation and the adjective to be on a Likert 1–6 scale. The WSAP-H showed good reliability (Cronbach’s alpha = 0.84, McDonald’s omega = 0.87). Scores were in accordance with the original norms [35]. As one could expect, hostile attributions were lower in the present community sample (mean = 2.72) than in Dutch men preselected for high hostility (mean = 3.42) [45].

The Sensitivity to Punishment and Sensitivity to Reward Questionnaire (SPSRQ) is comprised of 48 dichotomic items assessing reward seeking (e.g. “Do you sometimes do things for quick gains?”) and punishment avoidance (“Are you often afraid of new or unexpected situations?”) [37]. Reliability was good in the current sample (Cronbach’s alpha = 0.84, McDonald’s omega = 0.87). Mean values of both scales agreed with normative scores for adults [37]. There are no adult norms for the SPSRQ in Dutch-speaking countries.

Finally, we measured the lifetime frequency of specific risk-taking behaviours (e.g. “Drove 30mph or faster over the speed limit”) using the Risky, Impulsive, and Self-Destructive Behaviour Questionnaire (RISQ) [36]. Following authors’ recommendations, we created frequency bins to reduce skewness (0 = 0, 1–10 = 1, 11–50 = 2, 51–100 = 3, >100 = 4, >500 = 5). The instrument showed acceptable reliability in this study (Cronbach’s alpha = 0.78, McDonald’s omega = 0.84). There are no published Dutch norms for this questionnaire. Descriptive statistics for all questionnaires are provided in Table 1.

**Table 1:** Means (M) and standard deviations (SD) for all self-report scales (*n* = 256).

Questionnaire	M ± SD [Min-Max]
Aggression (BPAQ)	65.37 ± 16.51 [29–145]
Psychopathy (SRP-SF)	45.45 ± 12.62 [28–140]
Hostile attributions (WSAP-H)	2.72 ± 0.68 [1–6]
Benign attributions (WSAP-H)	4.13 ± 0.71 [1–6]
Punishment sensitivity (SPSRQ)	11.57 ± 5.18 [0-24]
Reward sensitivity (SPSRQ)	14.91 ± 4.23 [0-24]
Risky behaviors (RISQ)	2.64 ± 1.55 [0-5]

*BPAQ* Buss-Perry Aggression Questionnaire, *SRP-SF* Self-Report Psychopathy Scale-Short Form, *WSAP-H* Word Sentence Association Paradigm-Hostility, *SPSRQ* Sensitivity to Punishment and Sensitivity to Reward Questionnaire. *M* mean, *SD* standard deviation, *Min* Minimum, *Max* Maximum.

### Behavioural and self-report data analysis

For the HEX task, we tested whether participants shot more often and faster when gun probability was high and/or in a threat context. To do so, we ran a repeated-measures analyses of variance (ANOVA) with within-subject factors Gun probability (high, low) and Threat (high, low) on the percentage of shoot decisions and reaction times. These analyses were based on all *n* = 269 participants who correctly completed the task. For the HIBT, we also ran a repeated-measures ANOVA with factors emotion (anger, disgust, fear, and joy) and intensity (1 to 5) on the percentage of hostile responses and reaction times. We discarded the eight neutral faces to have the same number of faces in each category. These analyses were based on *n* = 251 participants with complete data.

We next inspected for a potential latent hostility factor common to both HEX and HIBT tasks. Since the sizeable multicollinearity precluded a reliable exploratory factor analysis, we adopted a two-stage approach: first, we ran a principal component analysis (PCA) on all task scores to uncover the latent structure of the data; second, we defined a structural equation model (SEM) to extract error-free scores for the identified latent factors, following previous work [46]. The PCA revealed that the data were most parsimoniously accounted for by a two-factor solution (see Fig. S3), each corresponding to one task. We then fit a SEM with a latent factor derived from the four HEX task conditions and a factor stemming from the four HIBT conditions, and we extracted individual scores on these latent factors to be used in subsequent analyses. Next, we correlated average shoot percentage in each condition of the HEX task (high and low gun probability and high and low threat) with the percentage of hostile appraisals for each emotion in the HIBT (anger, disgust, fear, and joy) for exploratory purposes (*n* = 207). A False Discovery Rate (FDR) threshold of *q* < 0.05 was applied to this group of comparisons to control for multiple testing. Finally, correlations were computed between questionnaire scores and the two task-derived latent factor loadings (*n* = 192) (for a similar procedure see [46]). These tests were FDR corrected at *q* < 0.05 as well. We also tested for gender differences in self-reports and behavioural performance by means of independent-samples t-tests.

Bayes Factors (BF) for the alternative hypothesis computed with default flat priors are reported alongside standard *p*-values. Effects were flagged as significant if *both* indices (*p* < 0.05, and BF > 3) supported the alternative hypothesis [12]. All analyses described in this section were performed using the ezANOVA package (https://cran.r-project.org/web/packages/ez/index.html) as well as the built-in stats package running on R version 4.0.5 [47] and R Studio version 1.4.1106 [48]. BFs were calculated using the BayesFactor package (https://cran.r-project.org/web/packages/BayesFactor/index.html).

### Computational modelling

**Fig. 3:**
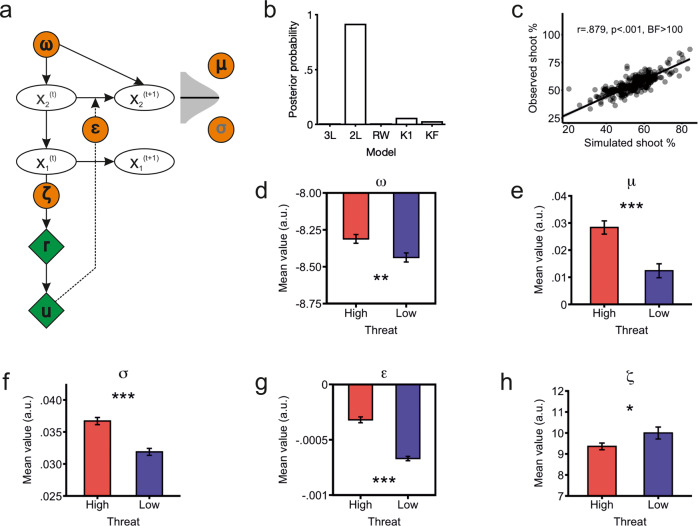
Computational modeling results. **a** Schematic depiction of the two-level Hierarchical Gaussian Filter (HGF) model. The first level (X_1_) represents the likelihood at trial *t* that a hostile outcome *u* (i.e., a gun) will ensue, whereas the second level (X_2_) encompasses the current perceived rate of hostile outcomes. Within this model there were five free parameters of interest estimated from participants’ responses *r* (i.e., shoot or withhold): volatility *ω* (speed of belief updates), mean second-level belief μ (average expected likelihood of hostile outcomes), uncertainty parameter *σ* (accuracy of the belief), precision-weighted prediction errors *ε* (contrast between expected and observed outcomes), and exploration parameter *ζ* (propensity to switch responses and thus deviate from the current belief level). Green diamonds represent the observed inputs (responses *r* and stim*u*li *u*), orange circles depict the five free parameters (*ω*, μ, *σ*, *ε*, and *ζ*) estimated from the inputs, and white ovals correspond to the model’s output, namely, trial-wise values of the current first- (X_1_) and second-level beliefs (X_2_). **b** Model comparison results. 3 L: 3-level HGF, 2 L: 2-level HGF, RW: Rescorla-Wagner, K1: Sutton K1 model, KF: Kalman Filter. **c** The winning model could successfully reproduce participants’ behaviour. **d** Mean volatility estimates *ω* were higher under high relative to low threat. **e** Mean value of the second-level belief *μ* parameter was higher under high relative to low threat. **f** Mean value of the second-level uncertainty parameter *σ* was higher under high relative to low threat. **g** Mean precision-weighted prediction errors *ε* were reduced under high relative to low threat. **h** Mean exploration readiness parameter *ζ* was reduced under high relative to low threat. *p < 0.05, BF Bayes Factor < 1; **p < 0.01, BF > 3; ***p < 0.001 and BF > 100.

The first goal was to test which reinforcement learning algorithm could best account for HEX learning. Drawing on previous work [3, 27], the following models were defined: a Rescorla-Wagner model, a K1 Sutton model, a Kalman filter [49] as well as 3- and 2-level Hierarchical Gaussian Filter (HFG) models [50]. All models were fitted using the HGF toolbox (https://tnu.ethz.ch/tapas), which estimates parameters using a Variational Bayes approach. This method shows a comparable performance to other popular optimization procedures such as the Nelder-Mead simplex algorithm or Markov Chain Monte Carlo estimation [50]. We used default priors to avoid biasing model selection, and compared models using Bayesian model selection [51], as implemented in the Statistical Parametric Mapping 12 toolbox (https://www.fil.ion.ucl.ac.uk/spm/) running on Matlab 2017b. A 2-level HGF was the best-fitting model (see Fig. 3a and b). The winning model was fitted again using the wider priors employed by Brazil et al. (2017) to better capture interindividual variability (note we had not altered the priors before to avoid influencing model selection). Next, a belief trajectory was simulated per participant using the final estimated parameters as priors. We inspected for correlations between estimated and simulated parameters as well as between actual and simulated percentage of shoot decisions to test how well the model could reproduce the real data.

The 2-level HGF for binary outcomes used here assumes that first-level beliefs $x_1^t$ reflect the estimated likelihood a given outcome (in this case, pulling a gun vs. a phone) at time *t*. The probability is $x_1^t$ is given by a Bernoulli distribution mapped to the current second-level belief $x_2^t$:

$$p(x_1^t = gun)\sim Bernoulli(x_1^t;s\left( {x_2^t} \right))$$

$$p(x_1^t = phone)\sim Bernoulli(x_1^t;1 - s\left( {x_2^t} \right))$$

Where $s\left( {x_2^t} \right)$ is a sigmoid transform of current second-level belief $x_2^t$:

$$s\left( {x_2^t} \right): = \frac{1}{{1 + {{{\mathrm{exp}}}}( - x_2^t)}}$$

Second-level beliefs $x_2^t$ are drawn from a Gaussian random walk defined by its own previous value, and its step size (i.e., its variance) is mapped to the exponentiated volatility parameter *ω*:

$$x_2^t\sim N(x_2^{t - 1},\exp \left( \omega \right))$$

As new inputs *u* are observed, the value of *x* is given by the following Gaussian posterior:

$$x|u\sim N(\mu _{x|u},\pi _{x|u}^{ - 1})$$

These quantities are then updated as follows:

$$\mu _1^t = \left\{ {\begin{array}{l} {1\;if\;u = gun} \\ {0\;if\;u = phone} \end{array}} \right.$$

$$\mu _2^t = \mu _2^{t - 1} + \varepsilon ^t$$

with

$$\varepsilon ^t = \frac{1}{{\pi _2^t}}\delta ^t$$

$$\pi _2^t = \hat \pi _2^t + \frac{1}{{\hat \pi _1^t}}$$

where

$$\hat \mu _1^t: = s\left( {\mu _2^{t - 1}} \right)$$

$$\delta ^t: = \mu _1^t - \hat \mu _1^t$$

$$\hat \pi _1^t: = \frac{1}{{\hat \mu _1^t(1 - \hat \mu _1^t)}}$$

$$\hat \pi _2^t: = \frac{1}{{\sigma + e^\omega }}$$

Higher values of *μ* thus reflect a stronger belief that the next outcome will be a gun. Prediction errors are $\delta ^t$ at the first level (discrepancy between current belief and prediction), and $\varepsilon ^t$ at the second ($\delta ^t$ weighted by precision estimate 1/$\pi _2^t$). Parameter *π* reflects the precision of the mean or prediction, and is determined by mean beliefs at the first level and by the variance (i.e., uncertainty) parameter *σ* at the second. Predictions $\hat \mu _1^t$ stem from a sigmoid transform of mean second-level beliefs in the previous trial, i.e., $s\left( {\mu _2^{t - 1}} \right)$. Finally, predictions are converted to action probabilities with a unit square sigmoid function to allow for decision noise, which is captured by the free parameter *ζ*:

$$p\left( {shoot} \right) = \left( {\frac{{\hat \mu _1^\zeta }}{{\hat \mu _1^\zeta + (1 - \hat \mu _1)^\zeta }}} \right)^\zeta + \left( {\frac{{(1 - \hat \mu _1)^\zeta }}{{\hat \mu _1^\zeta + (1 - \hat \mu _1)^\zeta }}} \right)^\zeta$$

Within this model we were fundamentally interested in five free parameters that indexed different aspects of HEX learning, namely: *ω*, *μ*, *σ*, *ε*, and *ζ*. These are detailed in Table 2 (along with an illustrative example) and depicted in Fig. 3. We tested whether the mean value of these parameters differed between the high- and low-threat conditions using paired *t*-tests.

**Table 2:** Parameters of interest from the winning model.

Parameter	Interpretation
*ω*	Belief updating as changes occur
*μ*	Mean belief in hostile outcomes
*σ*	Uncertainty/inaccuracy surrounding the belief
*ε*	Discrepancy between expected and actual outcome
*ζ*	Exploration readiness

The parameters can be exemplified as follows. Imagine that a person goes often to a bar where brawls happen frequently. An individual with higher ω values would be quicker to assume a decrease in the level of danger when there are no fights, whereas lower ω values would indicate perseverance in expecting brawls. Larger *μ* values would indicate a higher estimated fight rate. Uncertainty parameter σ would index the accuracy of this perception, such that a person with higher σ values would be more unsure about the overall brawl likelihood. Higher ε values would indicate stronger surprise when there is no fight. Finally, exploration readiness parameter ζ underlies how much a person’s responses deviate from the estimated brawl likelihood, i.e., whether their behavior explores options inconsistent with the inferred danger level.

### Structural equation modelling

Following Brazil et al. (2017), we inspected whether latent learning parameters were associated with antisocial behaviour and personality traits using structural equation modelling (SEM). Antisocial traits were expected to be associated with reduced volatility estimates, higher mean hostile beliefs, elevated variance/uncertainty in such estimates, larger prediction errors. Variables were converted to *z*-scores to normalize the data before running the analyses. The four aggression subscales were defined to load on a latent trait aggression factor, which was regressed on average participant-wise *ω*, *μ*, *σ*, *ε*, and *ζ* values in high threat blocks. The same model was fitted with mean parameter values from low threat blocks to test whether links between antisocial traits and learning parameters were general or rather condition-specific. We proceeded identically for psychopathy, namely, the four psychopathy subscales were loaded on a general latent factor [52], which was regressed on model-derived parameters separately for high and low threat blocks. Because trait aggression and psychopathic traits were highly correlated (*r* = 0.639, see Fig. S4), two alternative SEMs were tested: one with correlated psychopathy and aggressiveness factors and one in which all aggression and psychopathy subscales were loaded onto a single factor. The computational parameters were regressed on the latent variables in each model.

All SEM analyses were based on the *n* = 188 participants from whom we had complete HGF and self-report data. To examine the robustness of our findings, both standard estimation using lavaan [53] and Bayesian estimation using blavaan [54] were performed on R version 4.0.5 [47] and R Studio version 1.4.1106 [48]. Standard estimates were obtained with lavaan’s default maximum likelihood estimator, whereas Bayesian estimates were produced using blavaan’s Markov Chain Monte Carlo estimator with 5 chains at 10,000 samples each (the first 5000 samples were discarded as burn-in trials) and default wide normal priors (mean = 0, standard deviation = 10). Model fit indices and parameter estimates are reported for both methods. As fit measures we provide chi-square ($\chi ^2$, cut-off for good fit *p* > 0.05), Comparative Fit Index (CFI, cut-off for good fit > 0.95), root mean squared error of approximation (RMSEA, cut-off for good fit < 0.05) and root mean squared residual (SRMR, cut-off for good fit < 0.08) as recommended in SEM guidelines [55], as well as the Bayesian Posterior Predictive *p*-values (PPP, cut-off for good fit > 0.15, best fit when near 0.50) [56]. We report standardized regression coefficients and factor loadings alongside 95% Credible Intervals (CrI) resulting from Bayesian estimation as well as *p*-values derived from classical SEM. A result was considered statistically significant when the 95% CrI did not include zero and the *p*-value was below 0.05, i.e., only when *both* approaches yielded converging results [12].

## Results

### Task performance

#### Hostile expectation (HEX) learning task results

We first inspected participants’ performance in the HEX learning task. There were main effects of gun probability (F_1,268_ = 74.37, *p* < 0.001, BF > 100) and threat (F_1268_ = 16.92, *p* < 0.001, BF > 100) which were qualified by an interaction between the two factors (F_1,268_ = 10.94, *p* < 0.001, BF > 100). As shown in Fig. 1c, participants shot most frequently under high (59.71% ± 0.86% [mean ± standard error]) relative to low threat (55.29% ± 0.87%) when gun probability was high (t_268_ = 5.36, *p* < 0.001, BF > 100). Nevertheless, they shot at near-chance level irrespective of the threat condition (high threat: 52.28% ± 0.82%, low threat: 51.03% ± 0.88%) when gun probability was low (t_268_ = 1.46, *p* = 0.144, BF = 0.019).

Regarding reaction times, there was no conclusive evidence for effects of gun probability (F_1268_ = 21.68, *p* < 0.001, BF = 0.10), threat (F_1268_ = 0.41, *p* = 0.521, BF = 0.06), or an interaction (F_1268_ = 6.88, *p* < 0.001, BF < 0.01). This pattern was confirmed by post-hoc *t*-tests, which revealed only marginally faster reaction times when gun probability was low (t_268_ = 2.02, *p* = 0.043, BF = 0.511) and no difference when it was high (t_268_ = 1.20, *p* = 0.206, BF = 0.150). Thus, despite the apparently large *F* values, Bayesian analyses indicated that there were no substantial differences between conditions in reaction times (see Fig. S2).

### Hostile interpretation bias task (HIBT) results

In the HIBT, there were main effects of emotion (F_3,750_ = 275.94, *p* < 0.001, BF > 100), intensity (F_4,1000_ = 94.30, *p* < 0.001, BF > 100) and their interaction (F_12,3000_ = 100.29, *p* < 0.001, BF > 100). As shown in Fig. 2b, participants judged angry faces to be the most hostile as function of intensity, followed by those showing disgust, fear, and joy. There were also differences between categories in reaction times, with main effects of emotion (F_3750_ = 16.69, *p* < 0.001, BF > 100), intensity (F_4,1000_ = 38.91, *p* < 0.001, BF > 100) and, again, the interaction between both (F_12,3000_ = 5.87, *p* < 0.001, BF > 100). Participants were quickest to judge happy faces, followed by angry, disgusted, and fearful ones (see Fig. S5).

### Correlations between task-based measures of hostile biases

We inspected for linear relationships between hostile biases as assessed with the HEX and HIBT tasks (see Fig. S4 for all within- and between-task correlations). There were positive correlations between total percentage of shoot decisions and hostile interpretations of angry (r = 0.207, *p* = 0.002, *q* = 0.005, BF = 13.12) and disgusted facial expressions (r = 0.188, *p* = 0.006, BF = 5.88), but not of happy (*r* = 0.040, *q* = 0.643, *p* = 0.557, BF = 0.19) or fearful ones (*r* = 0.065, *q* = 0.421, *p* = 0.346, BF = 0.24). The strongest correlation was between shoot decisions under high threat and hostile appraisals of angry faces (r = 0.238, *p* < 0.001, *q* = 0.001, BF = 54.49). Most crucially, the two latent hostility factors were positively correlated (*r* = 0.238, *p* < 0.001, BF = 54.98; Fig. 2c).

### Correlations between latent hostile factors and self-reported antisocial tendencies

The latent factor derived from the HEX learning task was positively correlated with self-reported aggressiveness (*r* = 0.201, *p* = 0.004, *q* = 0.011, BF = 7.72) and psychopathy (*r* = 0.178, *p* = 0.013, *q* = 0.026, BF = 3.25), but not with hostile or benign attributions in ambiguous situations, sensitivity to punishment or reward, or risk-taking (all *r* < 0.146, *p* > 0.042, *q* > 0.072, and BF < 1.24; see Fig. S6 for all correlations between task scores and self-reports). The latent factor derived from the HIBT task was associated with lower punishment sensitivity (*r* = −0.189, *p* = 0.008, *q* = 0.019, BF = 4.84), more hostile attributions in ambiguous scenarios (*r* = 0.246, *p* < 0.001, *q* = 0.001, BF = 53.91), and marginally lower benign attributions (*r* = −0.165, *p* = 0.021, *q* = 0.038, BF = 2.18), but not with other self-report measures (all r < 0.116, *p* > 0.108, *q* > 0.163, and BF < 0.68). The HEX-derived factor was thus more closely linked with overt manifestations of antisocial behaviour (aggression and psychopathy), whereas the one extracted from HIBT scores was linked with social-cognitive (hostile attributions) and motivational (reduced punishment sensitivity) factors associated with aggressive and psychopathic traits. Hence, the observed pattern of associations generally supports the validity of the HEX and HIBT tasks.

### Gender differences in task performance and self-reports

As reported in Table S1, men had higher scores than women in the latent HEX factor (*t* = 3.90, *p* < 0.001, BF = 38.79), likely reflecting more shoot decisions across conditions. Nonetheless, there were no gender differences in hostile interpretations of human faces as indexed by the latent factor derived from the HIBT task (*t* = 0.28, *p* = 0.779, BF = 0.17). Men scored higher than women in self-reported aggression (*t* = 2.92, *p* = 0.003, BF = 8.72) and psychopathy (*t* = 4.49, *p* < 0.001, BF > 100), whereas women displayed more benign attributions in ambiguous scenarios (*t* = 2.74, *p* = 0.006, BF = 7.05). Men also reported to engage in more risk-taking behaviours than women (*t* = 2.72, *p* = 0.007, BF = 4.54). Women and men did not differ in hostile attributions, or in punishment or reward sensitivity (all *p* > 0.039 and BF < 1.32).

### Computational modelling results

A 2-level Hierarchical Gaussian Filter (HGF) model (Fig. 3a) provided the best fit to the data (Fig. 3b). Although the HGF model family had more free parameters than the other models, the finding that the winning model was a 2- rather than a 3-level HGF speaks against overfitting. We ran simulations with each participant’s mean parameter estimates as priors to assess how well the 2-level HGF could reproduce the observed values. The total percentage of simulated and observed shoot decisions were highly correlated (*r* = 0.879, Fig. 3c). The average correlation between estimated and simulated parameter trajectories were *r* = 0.896 ± 0.006 for the *μ* parameter, *r* = 0.997 ± 0.001 for the *σ* parameter, and *r* = 0.933 ± 0.003 for the *ε* parameter, suggesting excellent parameter recovery (see also Fig. S7). Simulated parameters *ω* and *ζ* converged on their prior and thus showed a perfect correlation with their estimated values.

We subsequently extracted average estimated parameter values from the high and low threat conditions and compared them with paired t-tests. Participants evinced greater (i.e., less negative) volatility *ω* (t_240_ = 6.49, *p* < 0.001, BF > 100; Fig. 3d), higher mean beliefs *μ* (t_240_ = 6.49, *p* < 0.001, BF > 100; Fig. 3e), increased uncertainty *σ* (t_240_ = 6.55, *p* < 0.001, BF > 100; Fig. 3f) and stronger (i.e., less negative) precision-weighted prediction errors in high relative to low threat (*ε*, t_240_ = 12.25, *p* < 0.001, BF > 100; Fig. 3g). High threat also elicited lower exploration readiness *ζ*, albeit the difference was substantially smaller than for all other parameters and not supported by the Bayesian analysis (t_240_ = 2.21, *p* = 0.027, BF = 0.799; Fig. 3h). Of note, there were no differences between women and men in HGF-derived parameters (all *p* > 0.281, all BF < 0.27; Table S2).

### Structural equation modelling results

**Fig. 4:**
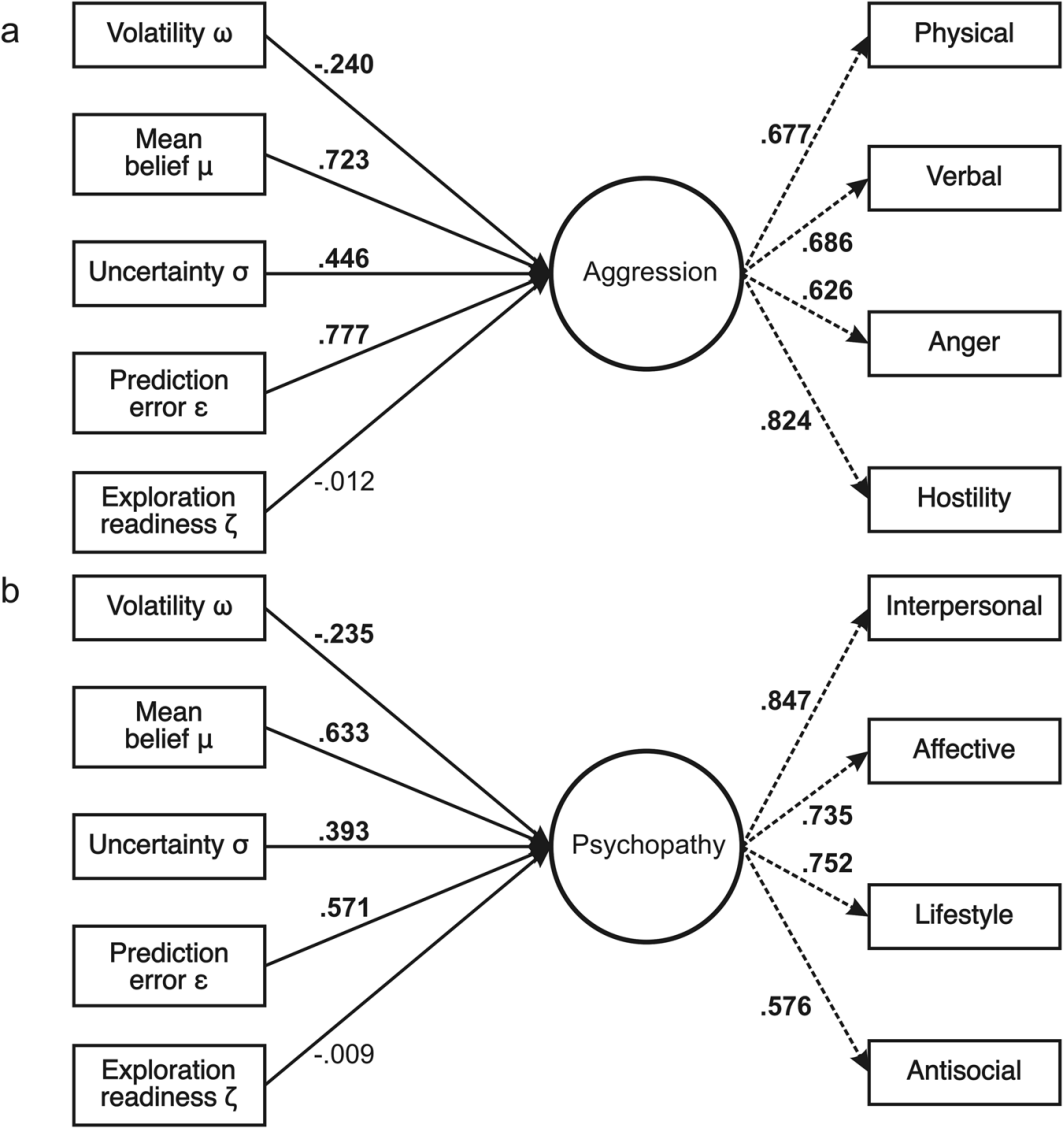
Structural equation modeling results. **a** Linear relationships between the latent aggression factor and learning parameters in high threat blocks (left) and loadings of the aggression factor on its four observed facets (right). **b** Linear relationships between the latent psychopathy factor and mean learning parameters in high threat blocks (left) and loadings of the psychopathy factor on its four observed facets (right). Solid lines denote regression coefficients, dashed lines denote factor loadings, and bold numbers denote statistically significant associations between variables (*p* < 0.05 and 95% Bayesian Credible Interval not including zero).

Next, we inspected for relationships between learning-related parameters in high threat blocks and the latent aggressiveness factor (Fig. 4a). The resulting SEM had excellent fit to the data (Frequentist: $\chi _{17}^2$ = 11.846, *p* = 0.809, CFI = 1, RMSEA < 0.001, SRMR = 0.024 / Bayesian: PPP = 0.720) and converged successfully (see Fig. S8 and Fig. S9). Aggressiveness was associated with lower volatility values *ω* (B = −0.240, *p* = 0.014, 95% CrI = [−0.445, −0.045]), greater mean beliefs *μ* (B = 0.723, *p* = 0.001, 95% CrI = [−0.489, −0.087]), higher uncertainty estimates *σ* (B = 0.446, *p* < 0.001, 95% CrI = [0.245, 767]), and greater second level precision-weighted prediction errors *ε* (B = 0.777, *p* = 0.001, 95% CrI = [0.405, 1.382]), but not with the exploration parameter *ζ* (B = −0.012, *p* = 0.838, 95% CrI = [−0.137, 0.105]). Classical (i.e., non-Bayesian) regression coefficients were highly similar to Bayesian ones and are reported in Table S3. We observed no associations between the latent aggressiveness factor and average learning parameters in the low threat condition (all *p* > 0.156 and all 95% CrI including 0; see Table S4).

We then tested whether the same learning-related parameters showed associations with the latent psychopathy factor (Fig. 4b). The model fit the data excellently (Frequentist: $\chi _{17}^2$ = 16.157, *p* = 0.513, CFI = 1, RMSEA < 0.001, SRMR = 0.024 / Bayesian: PPP = 0.546) and converged satisfactorily (see Fig. S10 and Fig. S11). Psychopathy was related to lower volatility estimates *ω* (B = −0.235, *p* = 0.044, 95% CrI = [−0.484, −0.003]), higher mean beliefs *μ* (B = 0.633, *p* = 0.010, 95% CrI = [0.127, 1.141]), increased uncertainty estimates *σ* (B = 0.393, *p* = 0.007, 95% CrI = [0.097, 712]), and higher second-level precision-weighted prediction errors *ε* (B = 0.571, *p* = 0.036, 95% CrI = [0.012, 1.138]). Psychopathic traits also failed to show an association with the exploration readiness parameter *ζ* (B = −0.009, *p* = 0.893, 95% CrI = [−0.155, 0.141]). Again, Bayesian and frequentist estimates were similar (Table S5). There were no relationships between the latent psychopathy factor and mean parameter values in the low threat condition (all *p* > 0.294 and all 95% CrI including 0, see Table S6).

Alternative models had substantially worse fit, i.e., one with correlated latent aggressiveness and psychopathy factors (Frequentist: $\chi _{49}^2$ = 106.285, *p* < 0.001, CFI = 0.909, RMSEA = 0.079, SRMR = 0.044 / Bayesian: PPP = 0.001) and one with a general antisocial factor (Frequentist: $\chi _{55}^2$ = 151.407, *p* < 0.001, CFI = 0.848, RMSEA = 0.097, SRMR = 0.054 / Bayesian: PPP < 0.001). We did thus not inspect these models further.

Sensitivity analyses showed that the main results held when rerunning the analyses with 10 additional participants who missed up to 50% of trials (see Table S7 and Table S8). Additionally, we fit the same SEMs to women and men separately to inspect for potential gender differences in the associations of aggressive and psychopathic traits with computational parameters in high threat blocks (see Tables S9 to S12 for complete results). In women, all effects remained significant ( | B | = [0.426, 0.720], all *p* < 0.006 and 95% CrI not including zero) except for volatility ω, which was not associated with either trait aggression (B = −0.170, *p* = 0.070, 95% CrI = [−0.366, 0.022]) or psychopathy (B = −0.138, *p* = 0.240, 95% CrI = [−0.381, 0.097]). In men, effects of learning parameters on aggressiveness failed to reach significance (all *p* > 0.101) but were medium in size ( | Bs | =[0.370, 0.515]), whereas associations between these same parameters and trait psychopathy were substantially lower ( | Bs | = [0.003, 0.424]). Regression coefficients for exploration readiness *ζ* remained non-significant in all models ( | Bs | = [0.036, 0.179], all *p* > 414, 95% CrI including zero). Factor loadings of each questionnaire subscale on the corresponding construct (aggression or psychopathy) remained substantial across subsamples ( | B | = [0.553, .907], all *p* < 0.001 and 95% CrI not including zero). Finally, post-hoc power analyses for the structural equation models revealed over 92% (aggression model) and 98% power to falsify the fitted models in the event they were misspecified (see Power Analyses in Supplementary Material).

## Discussion

In the present study we set out to test whether hierarchical reinforcement-learning could account for the acquisition of Hostile Expectations (HEX) in a task that required participants to decide between aggressive and non-aggressive actions. We also addressed whether HEX were more easily learned in a high- relative to a low-threat context [41]. Finally, we inspected for relationships between HEX and other forms of antisocial behaviour and cognition. Specifically, we expected that aggressive and psychopathic traits would be associated with higher and more uncertain hostile beliefs, slower HEX updating (i.e., lower volatility), and more pronounced prediction errors. There were three key findings. First, HEX learning was best captured by a Bayesian learning model that incorporated both specific outcome expectations and their overall likelihood. Therefore, individuals did not merely come to expect hostile outcomes but also estimated the rate at which they ensued. Second, threat facilitated HEX learning by virtue of stronger, increasingly variable, and more volatile beliefs as well as through increased prediction errors. Hence, a threatening context seems to induce more imprecise and malleable hostility representations, which facilitates aggressive responding. Third, aggressive and psychopathic personality traits were linked with stronger and more uncertain hostile beliefs, increased prediction errors, and reduced volatility estimates. These results supported our hypotheses, demonstrating that individuals with higher levels of aggression and/or psychopathy are likely to develop solid but inexact hostility beliefs which then become resistant to change.

Our findings indicate that participants shot more often in the high relative to the low threat condition. This observation agrees with previous studies using the shooting task, wherein participants were more accurate under the prospect of punishment [40, 41]. However, in these investigations participants did not need to predict the opponent’s behaviour but had to *react* to it, in line with other studies reporting a threat-boosted improvement in accuracy in reaction time tasks [57–59]. Here, we show that this effect extends to instrumental learning, so that the presence of threat ultimately improves the prediction of hostile behaviour. Computational modelling attributed this pattern to elevated, uncertain, and quickly evolving hostility representations under high relative to low threat, as well as stronger surprise when these beliefs were contradicted. In the following we comment on each of these findings.

We found that the presence of threat led to quicker belief updates (i.e., higher volatility estimates) in response to changes in the environment. Our results parallel previous reports of faster belief updating when forming moral impressions of antisocial individuals -who constitute a potential threat- relative to altruistic ones [3, 60]. The present findings further show that swift updating of beliefs is not only adaptive when dealing with threatening individuals, but also in potentially harmful contexts. Therefore, hostility representations are learned more quickly but are more malleable under high threat.

We also observed increased hostile beliefs in the high as compared to the low threat condition. In a high threat situation, this pattern can be interpreted as a proneness to overlearn from aversive events and thus establish hostile associations. Participants also formed more uncertain (i.e., less accurate) beliefs in high relative to low threat contexts. Higher uncertainty estimates improve associative learning as a function of pupil dilation and subjective stress, indicating that physiological arousal facilitates the acquisition of aversive associations [20]. The combination of elevated and uncertain hostility beliefs thus resulted in a more robust acquisition of hostile expectations, perhaps due to a threat-dependent increase in arousal and alertness. This notion is further supported by the finding that blocking the action of acetylcholine (a key neurotransmitter for arousal maintenance) disrupts learning under uncertainty by slowing belief updating and reducing prediction errors [61]. In contrast, in the present study high threat led to increased precision-weighted prediction errors, which reflect sensitivity to unexpected events and have been shown to engage midbrain dopaminergic structures assumed to underlie social and sensory learning [22, 62]. Therefore, a potentially threatening environment partly potentiates HEX acquisition by means of a greater sensitivity to changes in outcome probability. The threat manipulation also led to a slight decrease in exploration readiness, suggesting that participants engaged in less exploration under high threat, i.e., responded more consistently depending on the opponent’s perceived level of hostility. Note that this effect should be interpreted with caution as it was not supported by the Bayesian analysis. Still, such a notion concurs with the reduction in random responding observed when individuals learn probabilistic associations to benefit someone (themselves or others) as compared to when no money is at stake [63]. To sum up, a high threat context facilitates the acquisition of aggressive responses by eliciting stronger, more variable, and volatile hostility representations as well as an enhanced reactivity to mismatching feedback.

We further inspected for a common hostile bias across learning and perception using principal component analysis on scores from the HEX and Hostile Interpretation Bias task (HIBT). Aggressive individuals prioritize the processing of threatening over neutral information, allocating more attentional resources to these stimuli [64]. Such preferential threat processing has been proposed to partly underlie hostile intent attribution [30, 65], and to facilitate anger perception [66, 67]. It has been suggested that all hostile biases tap onto a common mechanism, which will be expressed differently depending on the cognitive process, be it perception (interpreting ambiguous sensory cues as hostile), prediction (expecting hostile outcomes in ambiguous situations), or attribution (assuming hostile intent behind neutral or ambiguous acts) among others [11]. Yet, because this proposal is relatively new and these phenomena have been studied in isolation, support for a unified hostile bias is still preliminary. Although a two-factor solution emerged from the analysis, the correlation between the two latent factors could be indicative of separate, albeit related hostile biases in the learning and perceptual domains. This dissociation is corroborated by the observation that more hostile and less benign attributions in ambiguous scenarios were only associated with the HIBT -but not the HEX- latent factor. The latent HIBT factor was further correlated with lower punishment sensitivity but not with aggressive or psychopathic traits, unlike previous research [34]. Rather, aggressiveness and trait psychopathy were most strongly linked with the latent HEX factor. These discrepant findings might be reconciled by the notion that aggressiveness can bias threat processing in some domains (e.g., excessively hostile appraisals of social situations [11]) but sharpen it in others (e.g., anger perception [66, 67]). Our results thus show that hostile tendencies in the perceptual and social-cognitive domains partly extend to the instrumental acquisition of aggressive responses during interpersonal confrontations. Future studies should test whether HEX acquisition also generalizes to other cognitive functions, such as memory (preferential encoding and retrieval of hostile information [68]) or perception in other sensory modalities (e.g., hostile interpretations of ambiguous vocalizations [69]). To that end, experimental designs could be optimized so that tasks are more readily comparable to each other. For instance, one could employ the same stimuli to measure different cognitive processes (e.g., finding hostile words in a list vs keeping the same list in working memory), or, complementarily, by measuring the same cognitive process with different stimuli (e.g., selective attention to auditory vs visual hostile cues).

We also uncovered multiple associations between the cognitive processes involved in HEX acquisition with psychopathic personality traits and aggression. First, aggressive and psychopathic traits covaried with less volatile hostility representations, in line with one of our main hypotheses. This finding suggests that the environment was estimated to remain more stable by participants as the level of trait aggression and psychopathy increased. It should be noted that volatility is beneficial to learning, as it allows for quicker adaptation [3]. Indeed, while volatility was higher in the high threat condition, aggressive and psychopathic traits were linked with *lower* volatility. Such a pattern is broadly consistent with the well-documented impairments in reinforcement-learning displayed by psychopathic individuals, who fail to flexibly adapt to changes in the environment [16–19]. This deficit might be partially due to an “exaggerated attentional bottleneck”, i.e., a tendency to distribute attentional resources suboptimally and filtering out too much relevant information when multiple streams of information need to be processed [70]. It is possible that the presence of an excessively active attentional bottleneck in individuals with stronger psychopathic tendencies could have hampered the monitoring of multiple sources of information in our task (i.e., the officer and the man), ultimately resulting in smaller belief updates regarding volatility as part of the information required for optimal belief updating is filtered out. Also, paranoid ideation -a well-known risk factor for aggression [30]- has been linked to lower volatility estimates during reversal learning, indicating more rigid beliefs [21]. State anxiety has been similarly reported to impair learning by reducing perceived volatility [26]. In contrast, volatility *over*estimation has been rather associated with internalizing traits such as autism [71], deficits in emotional coping [27], or chronic stress levels [20]. Therefore, externalizing symptomatology seems to be characterized by an insufficient adaptation to changes in the environment, whereas internalizing symptoms would instead be linked with excessive belief updating. In sum, the current results postulate volatility underestimation as a promising cognitive marker for aggressive and psychopathic personality profiles.

Second, we discovered that beliefs about hostility were stronger and less accurate in individuals scoring high in trait psychopathy and/or aggression, supporting our hypotheses. In line with our results, impulsive-irresponsible psychopathic traits have been positively associated with uncertainty representations in the insula and amygdala during threat conditioning [4]. In the present investigation, we complement these findings by showing that inaccuracy in the generated beliefs also potentiates instrumentally acquired aggressive behaviour in persons with relatively higher levels of aggression and psychopathy. Such a pattern might render aggressive and psychopathic individuals more prone to expect harm from others and thus respond in an indiscriminately aggressive manner to ambiguous cues. Supporting this tenet, psychopathic traits have been associated with enhanced risk-taking under uncertainty [38] and trait aggression correlates with more impulsive judgements of others’ hostility [72]. Similarly, a study showed that aggressive individuals more readily deliver electric shocks to persons whose face had been previously coupled with an aversive stimulus [73]. The latter observation suggests that aggressive and psychopathic traits ease the translation of acquired aversive associations into overt aggressive behaviour. Here, we extend this phenomenon to an instrumental learning context, so that individuals with higher levels of aggressiveness and psychopathy readily generalize aggressive responses in threatening social encounters. Though such a tendency might be adaptive when facing potentially harmful individuals, it might result in maladaptive behaviour in most other situations. Indeed, while a majority of studies reported learning deficits in antisocial individuals [16], our correlation analyses relate *better* HEX acquisition with higher levels of aggressiveness and psychopathy. Note that this benefit occurred despite the underestimation of environmental volatility observed as a function of trait aggression and psychopathy, which resulted in a more sluggish belief actualization. In other words, persons with high levels of aggressiveness and psychopathy formed more robust and imprecise hostility representations, and adapted less to changes in the environment. The present findings are consistent with the notion that learning in psychopathy is dominated by general valence encoding but inaccurate value estimation [74]. According to this view, psychopathic individuals can learn whether a cue predominantly predicts good or bad outcomes but fail to correctly approximate the true reinforcement or punishment rates. Here, we additionally show that aggressiveness similarly contributes to an inflexible consolidation of hostile beliefs.

Third, the discrepancy between expected and obtained outcomes (i.e., the prediction error) was larger as the level of aggressive and psychopathic traits increased in our sample, in accordance with our last hypothesis. This pattern reflects greater surprise to the violation of expectancies in individuals with high levels of these traits [61]. Previous studies have shown that aggressive individuals display enhanced surprise-like neural activity when HEX are violated [31, 32]. Our results agree with these reports and further show how such a heightened deviance monitoring facilitates hostility learning. These findings concur as well with the above-mentioned link between psychopathy and inaccurate associative learning. More recent proposals further suggest that individuals with high levels of psychopathy use relatively longer time windows to acquire stimulus-outcome associations [75]. Our results expand this view by showing that psychopathy is tied with a tendency to develop strong, noisy, and slowly-evolving associations in threatening contexts and thus show an enhanced sensitivity to hostile expectation violation.

It may be apparently puzzling that psychopathy and aggressiveness showed such a similar pattern of covariation with other variables, considering the differences between both constructs. Nevertheless, pooled evidence indicates a substantial association between psychopathy and aggression, which share features such as impulsivity or poor behavioural control [76, 77]. Indeed, although psychopathy has been traditionally linked to proactive or instrumental aggression, it is now clear that psychopathic traits also increase the risk for reactive or impulsive aggression [76–78]. Yet, the antisocial or criminal dimension of the psychopathy questionnaire was only weakly to moderately -though significantly- correlated with the verbal aggression (*r* = 0.14), hostility (*r* = 0.18), and anger (*r* = 0.23) scales of trait aggression (see Fig. S12 for all scale intercorrelations). This might partly explain why psychopathy and aggression did not load onto a single latent factor, despite the correlation between questionnaire-derived scores and the similar associations that both latent factors showed with learning-related variables. Our results therefore add to the literature by suggesting a computational substrate common to both aggressive and psychopathic tendencies, and point towards learning processes as a promising intervention target. A previous study suggested that interventions based on attention to context (e.g., reversal learning tasks) work best for individuals with psychopathy, whereas persons with high reactive aggression benefit most from affective cognitive control training (e.g., response inhibition tasks) [79]. Our findings tentatively suggest that reinforcement learning tasks using more explicit hostile cues might prove useful in the assessment and treatment of both aggressiveness and psychopathic traits. This could potentially improve not only the *valence* (i.e., less negative judgements) but also the *accuracy* (i.e., correctly estimating danger) of the social inferences made by individuals with high levels of psychopathy and trait aggression.

All in all, we were able to demonstrate that the acquisition of HEX can be linked to real-world indices of aggression and psychopathy. The generalizability of our findings is nonetheless curtailed by the use of a predominantly female community sample, which is not readily comparable to clinical or forensic populations, and of self-reports, which are notoriously affected by social desirability [80]. Thus, it should be determined whether the computational mechanisms outlined here can also aid the prediction of overt antisocial behaviour in applied settings using more extensive multi-method assessments, such as interviews or reports from close others. Notably, computational parameters did not differ by gender but were more strongly associated with aggressive and psychopathic traits in women than in men. However, the differing size of the female and male subsamples hinders gender comparisons. Our behavioural measures may also suffer from a lack of ecological validity. This is especially true for the HIBT paradigm, as its superimposed face images are less realistic than morphed expressions [66]. Moreover, the HIBT does not allow to disentangle bias from accuracy [66, 67]. Finally, we used a cross-sectional design and thus it is unclear how HEX are maintained across the lifespan. Longitudinal and developmental studies are required to characterize the long-term evolution of cognitive hostility biases [8] and how it is shaped by certain life events such as exposure to violence [60].

## Conclusion

Taken together, our results shed light on the architecture of hostile thought. We demonstrate that HEX acquisition can be understood as a reinforcement-learning process that is sharpened under high threat. Further, we report a link between instrumental learning of aggressive responses and hostile appraisals of human faces [11]. Finally, we show that aggressive and psychopathic traits are linked with the development of strong, imprecise, and temporally stable hostility beliefs as well as with increased sensitivity to events that deviate from such representations. Our findings thus provide a refined characterization of cognitive markers that might prove valuable in the understanding and prediction of antisocial behaviour.

## Supplementary information

Supplementary Material

## Data Availability

Open Science Practices: All data from this study are accessible to reviewers via the Donders Repository: 10.34973/bqmj-m165. A similar version of this manuscript is available as a preprint on PsyArxiv 10.31234/osf.io/2ydsz.
