# Supplementary material for: Aggressive and psychopathic traits are linked to the acquisition of stable but imprecise hostile expectations
Source: Transl Psychiatry. 2023 Jun 10;13:197. doi: 10.1038/s41398-023-02497-0 (PMC10256845; doi:10.1038/s41398-023-02497-0)
Supplement: Supplementary file 1 — Supplementary Material [file 41398_2023_2497_MOESM1_ESM.docx]

**Figure S1**. Overview of data exclusions


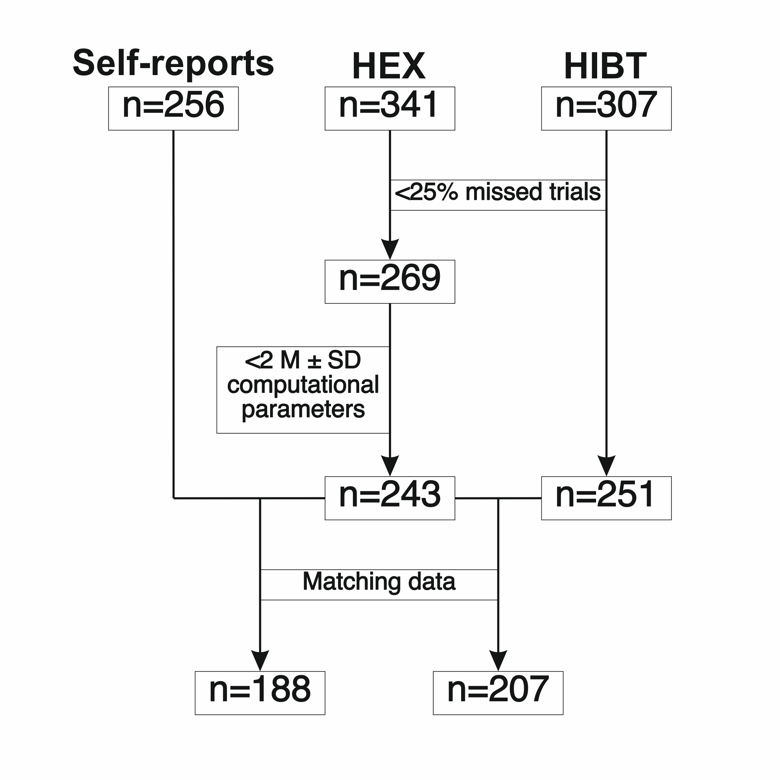


M: Mean, SD: Standard Deviation, HEX: Hostile Expectation task, HIBT: Hostile Interpretation Bias Task. Participants excluded due to outlying values in their computational parameters did not differ from the rest in age (t_28.19_=0.87, p=.390, BF=0.29), gender, ($\chi$^2^[2, *N*=210]=1.99, p=.920), handedness ($\chi$^2^[2, *N*=210]=4.61, p=.202), education ($\chi$^2^[4, *N*=210]=2.74, p=.600), or substance use (all p>.343). They were also comparable to the rest of the sample in trait aggression (t_24.40_=0.13, p=.896, BF=0.23), psychopathy (t_25.85_=0.60, p=.547, BF=0.27), hostile attributions of ambiguous scenarios (t_25.76_=0.05, p=.956, BF=0.23), benign attributions of the same scenarios (t_26.33_=1.50, p=.144, BF=0.60), punishment sensitivity (t_25.97_=0.13, p=.891, BF=0.23), reward sensitivity (t_25.26_=2.09, p=.046, BF=2.10), risk-taking behaviour (t_25.85_=0.64, p=.527, BF=0.28), and in hostile appraisals of facial expressions as indexed by the latent factor derived from the HIBT (t_19.31_=0.74, p=.467, BF=0.35; see *Behavioural and self-report data analysis* and Fig. S4). Excluded participants did show a trend toward reduced shoot percentage as measured by the latent HEX factor (t_17.34_=1.52, p=.145, BF=39.30), a difference driven by a particularly reduced tendency to shoot in low threat trials (t_17.52_=1.88, p=.076, BF=123.76; included: 53.57%$\pm$0.71%, excluded: 42.55%$\pm$5.80%).

**Figure S2**. Reaction times in the Hostile Expectation Learning (HEX) task as a function of threat and gun probability


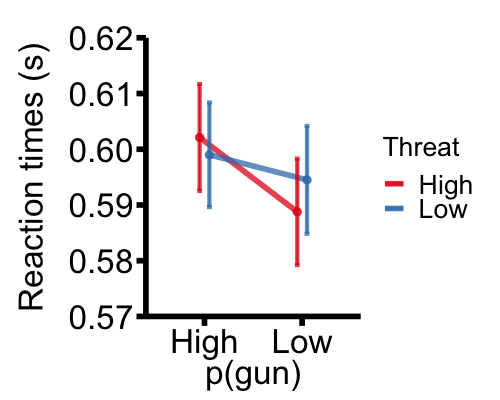


S: seconds. Values are mean $\pm$ standard error.

**Figure S3.** Results of the Principal Component Analysis

**
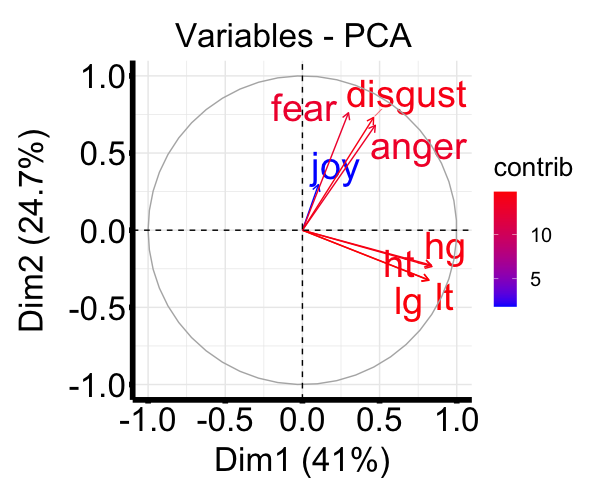
**

Results of the principal component analysis on scores extracted from the Hostile Expectation (HEX) learning task and the Hostile Interpretation Bias Task (HIBT). A two-factor solution accounted for 64.7% of the variance in observed scores and could accommodate well all indices except “joy”, which still loaded onto its theoretically expectable factor. hg: shoot % under high gun probability in the HEX task; lg: shoot % under low gun probability in the HEX task; ht: shoot % under high threat probability in the HEX task; lt: shoot % under low threat probability in the HEX task; anger: % hostile responses to angry faces in the HIBT; joy: % hostile responses to happy faces in the HIBT; fear: % hostile responses to fearful faces in the HIBT; disgust: % hostile responses to disgusted faces in the HIBT; htot: % total hostile responses to in the HIBT. Values indicate individual component loadings for each variable and are represented by arrows. n=207. These analyses were performed using the function *prcomp()* from the stats R package.

**Figure S4**. Correlations between scores in each condition of the HEX and HIBT tasks


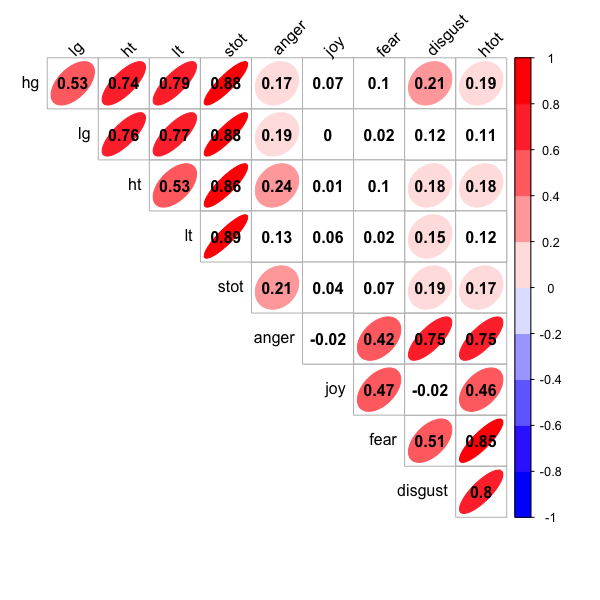


hg: shoot % under high gun probability in the Hostile Expectation (HEX) learning task; lg: shoot % under low gun probability in the HEX task; ht: shoot % under high threat probability in the HEX task; lt: shoot % under low threat probability in the HEX task; stot: average shoot % across all conditions probability in the HEX task; anger: % hostile responses to angry faces in the Hostile Interpretation Bias Task (HIBT); joy: % hostile responses to happy faces in the HIBT; fear: % hostile responses to fearful faces in the HIBT; disgust: % hostile responses to disgusted faces in the HIBT; htot: % total hostile responses to in the HIBT. Values are Pearson correlation coefficients; more intense hues and elongated ellipses depict larger values. Blank squares correspond to non-significant correlations after False Discovery Rate correction for multiple comparisons (q<.05). n=207.

**Figure S5**. Reaction times in the Hostile Interpretation Bias Task (HIBT) as a function of emotion and intensity


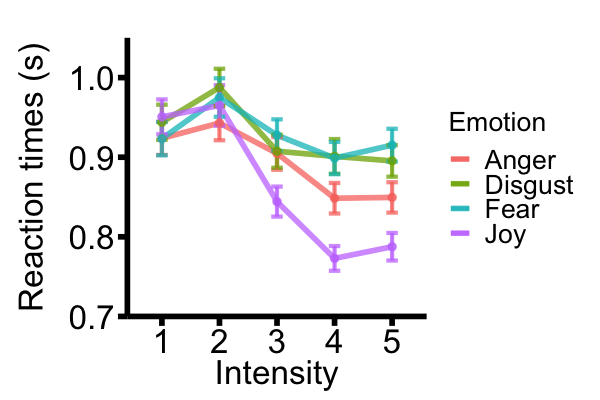


S: seconds. Values are mean $\pm$ standard error.

**Table S1**. Gender differences in behavioural measures and self-reports

| Variable | Women (M$\pm$SD, n=177) | Men (M$\pm$SD, n=79) | Statistics |
| --- | --- | --- | --- |
| **HEX (a.u.)** | **-0.16**$\boldsymbol{\pm}$**0.85%** | **0.26**$\boldsymbol{\pm}$**0.60%** | **t=3.90, p<.001, BF=38.79** |
| HIBT (a.u.) | 0.002$\pm$0.78 | 0.034$\pm$0.71 | t=0.28, p=.779, BF=0.17 |
| **Aggression** | **63.36**$\boldsymbol{\pm}$**16.1** | **69.87**$\boldsymbol{\pm}$**16.63** | **t=2.92, p=.003, BF=8.72** |
| **Psychopathy** | **43.01**$\boldsymbol{\pm}$**11.29** | **50.94**$\boldsymbol{\pm}$**13.76** | t=4.49, p<.001, BF>100 |
| Hostile attributions | 2.67$\pm$0.68 | 2.83$\pm$0.68 | t=1.78, p=.076, BF=0.65 |
| **Benign attributions** | **4.21**$\boldsymbol{\pm}$**0.67** | **3.94**$\boldsymbol{\pm}$**0.76** | **t=2.74, p=.006, BF=7.05** |
| Punishment sensitivity | 11.28$\pm$5.32 | 12.24$\pm$4.81 | t=1.43, p=.154, BF=0.36 |
| Reward sensitivity | 15.30$\pm$4.07 | 14.03$\pm$4.47 | t=2.07, p=.039, BF=1.32 |
| **Risky behaviours** | **2.46**$\boldsymbol{\pm}$**1.54** | **3.03**$\boldsymbol{\pm}$**1.52** | **t=2.72, p=.007, BF=4.54** |

HEX: latent factor derived from the Hostile Expectation learning task; HIBT: latent factor scores derived from the Hostile Interpretation Bias Task (HIBT). BPAQ: Buss-Perry Aggression Questionnaire, SRP-SF= Self-Report Psychopathy Scale-Short Form, WSAP-H: Word Sentence Association Paradigm-Hostility, SPSRQ: Sensitivity to Punishment and Sensitivity to Reward Questionnaire. M: mean, SD: standard deviation, n=group size, a.u.: arbitrary units, t=t-values, p: p-values, BF: Bayes Factor.

**Figure S6**. Correlations between self-report and behavioural measures


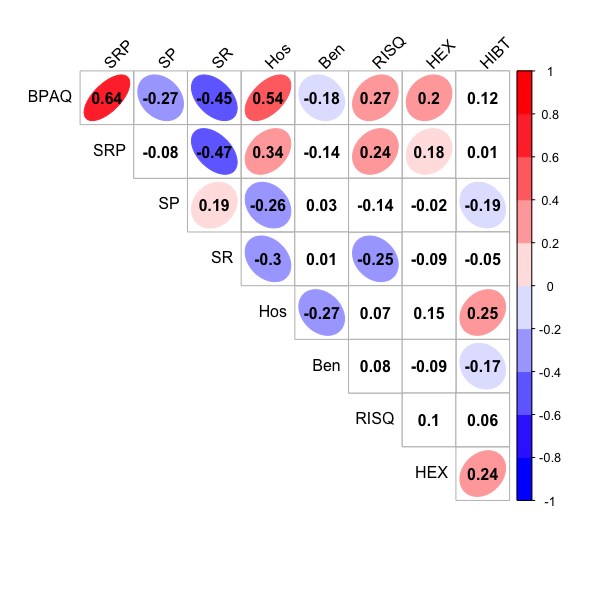


BPAQ: Buss-Perry Aggression Questionnaire, total score; PSY: Self-Report Psychopathy scale – Short Form, total score; SP: Sensitivity to Punishment scale from the Sensitivity to Punishment and Sensitivity to Reward Questionnaire (SPSRQ), SR: Sensitivity to Reward scale from the Sensitivity to Punishment and Sensitivity to Reward Questionnaire (SPSRQ); Hos: hostile attributions in ambiguous vignettes as measured with the Word Sentence Association Paradigm – Hostility (WSAP-H); Ben: benign attributions in ambiguous vignettes as measured with the Word Sentence Association Paradigm – Hostility (WSAP-H); RISQ: Risky, Impulsive, and Self-Destructive Behaviour Questionnaire (RISQ), total score (total lifetime number of risky behaviours); HEX: latent factor derived from the Hostile Expectation learning task; HIBT: latent factor scores derived from the Hostile Interpretation Bias Task (HIBT). Values are Pearson correlation coefficients; more intense hues and elongated ellipses depict larger values. Blank squares correspond to non-significant correlations after False Discovery Rate correction for multiple comparisons (q<.05). n= 192.

**Figure S7**. Parameter recovery of the model that best explained hostile expectation learning (a 2-level Hierarchical Gaussian Filter). Depicted are estimated (orange) and simulated (blue) second level belief trajectories for 10 example participants (see main body of the manuscript for a complete report of parameter recovery results).


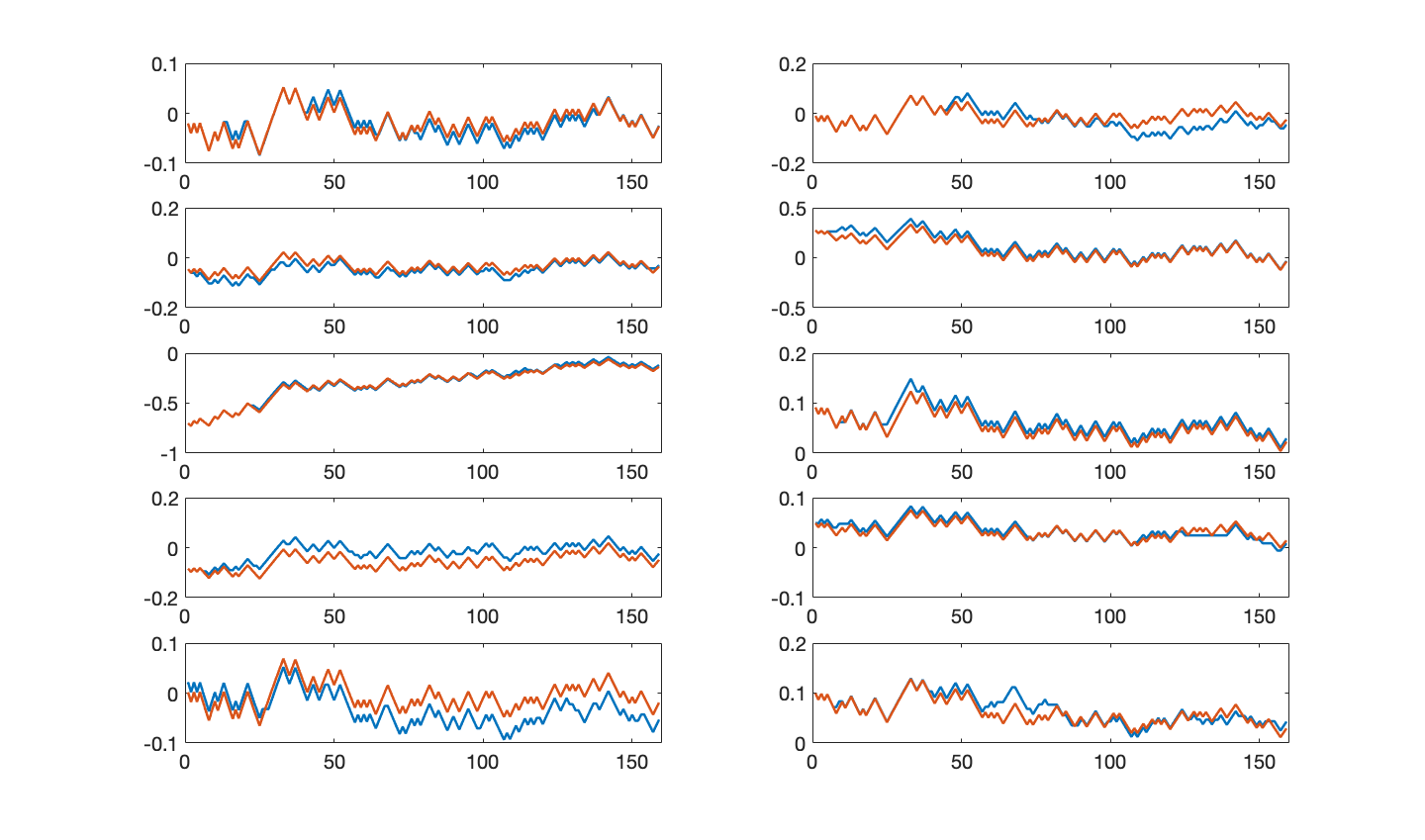


X axis displays trial number, Y axis displays the parameter value in arbitrary units.

**Table S2**. Gender differences in computational parameters derived from the 2-level Hierarchical Gaussian Filter winning model fitted to the HEX task

| Variable | Women (M$\pm$SD, n=124) | Men (M$\pm$SD, n=64) | Statistics |
| --- | --- | --- | --- |
| Volatility $\omega$ | -8.28$\boldsymbol{\pm}$0.48 | -8.30$\boldsymbol{\pm}$0.42 | t=-0.36, p=.712, BF=0.17 |
| Mean belief $\mu$ | 0.028$\boldsymbol{\pm}$0.036 | 0.034$\boldsymbol{\pm}$0.031 | t=1.08, p=.281, BF=0.27 |
| Uncertainty $\sigma$ | 0.037$\boldsymbol{\pm}$0.0088 | 0.036$\boldsymbol{\pm}$0.0082 | t=-0.43, p=.667, BF=0.18 |
| Prediction error $\varepsilon$ | -0.00032$\boldsymbol{\pm}$0.00042 | -0.00037$\boldsymbol{\pm}$0.00034 | t=-0.85, p=.393, BF=0.22 |
| Exploration readiness $\zeta$ | 9.27$\boldsymbol{\pm}$2.73503 | 8.97$\boldsymbol{\pm}$1.96 | t=-0.84, p=.399, BF=0.21 |

M: mean, SD: standard deviation, n=group size, a.u.: arbitrary units, t=t-values, p: p-values, BF: Bayes Factor.

**Figure S8**. Trace and autocorrelation plots for regression coefficients in the Bayesian structural equation model predicting the latent aggression factor (agg) from computational parameters in high threat blocks


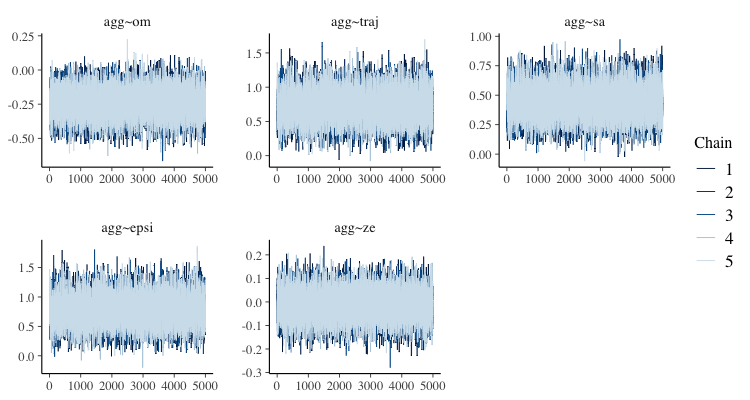


X axis depicts the iteration in each chain after burn-in, Y axis displays the value of the regression parameter. The overlap between chains shows convergence around the same values. om: volatility $\omega$, traj: mean belief $\mu$, sa: uncertainty $\sigma$, epsi: Prediction error ε, ze: exploration ζ.

**Figure S9**. Trace and autocorrelation plots for regression coefficients in the Bayesian structural equation model predicting the latent aggression factor from computational parameters in high threat blocks


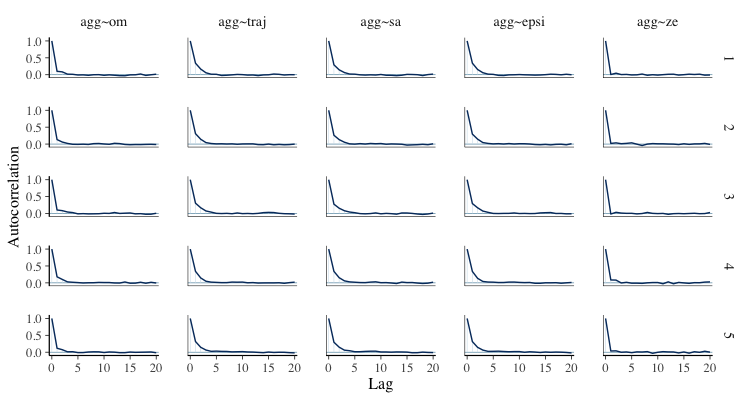


X axis shows the distance between current and past iterations, Y axis represents the correlation between those two iterations. Well-behaving chains are expected to fluctuate constantly (see the spread in the trace plot above) and thus show low autocorrelation with distant iterations, as is the case here. om: volatility $\omega$, traj: mean belief $\mu$, sa: uncertainty $\sigma$, epsi: Prediction error ε, ze: exploration ζ.

**Table S3**. Results of the structural equation model on latent trait aggression in high threat blocks

|  | Estimate | z | p |
| --- | --- | --- | --- |
| Factor loadings | | | |
| Physical aggression | .677 |  |  |
| Verbal aggression | .686 | 7.78 | <.001 |
| Hostility | .626 | 7.23 | <.001 |
| Anger | .824 | 8.52 | <.001 |
| Regression coefficients | | | |
| Volatility $\omega$ | -.238 | -2.45 | .014 |
| Mean belief $\mu$ | .719 | 3.47 | .001 |
| Uncertainty $\sigma$ | .441 | 3.54 | <.001 |
| Prediction error $\varepsilon$ | .771 | 3.33 | .001 |
| Exploration readiness $\zeta$ | -.012 | -0.20 | .838 |

Note that the first indicator (in this case, physical aggression) is fixed to one by default to set the scale of the latent variable and thus its p-value cannot be estimated. Z: z-score, p: p-value.

**Table S4**. Results of the structural equation model on latent trait aggression in low threat blocks

|  | Estimate | z | p |
| --- | --- | --- | --- |
| Factor loadings | | | |
| Physical aggression | .685 |  |  |
| Verbal aggression | .689 | 7.85 | <.001 |
| Hostility | .666 | 7.64 | <.001 |
| Anger | .778 | 8.39 | <.001 |
| Regression coefficients | | | |
| Volatility $\omega$ | -.120 | -0.85 | .395 |
| Mean belief $\mu$ | -.001 | -0.005 | .996 |
| Uncertainty $\sigma$ | .045 | 0.21 | .831 |
| Prediction error $\varepsilon$ | -.133 | -0.52 | .598 |
| Exploration readiness $\zeta$ | .117 | 1.42 | .156 |

Note that the first indicator (in this case, physical aggression) is fixed to one by default to set the scale of the latent variable and thus its p-value cannot be estimated. Z: z-score, p: p-value.

**Figure S10**. Trace plots for regression coefficients in the Bayesian structural equation model predicting the latent psychopathy factor (psy) in high threat blocks


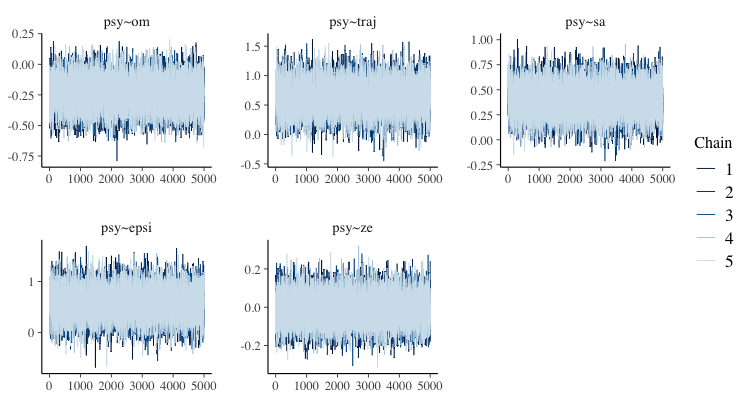


X axis depicts the iteration in each chain after burn-in, Y axis displays the value of the regression parameter. The overlap between chains shows that all chains converged around the same values. om: volatility $\omega$, traj: mean belief $\mu$, sa: uncertainty $\sigma$, epsi: Prediction error ε, ze: exploration ζ.

**Figure S11**: autocorrelation plots for regression coefficients in the Bayesian structural equation model predicting the latent psychopathy factor in high threat blocks from computational parameters


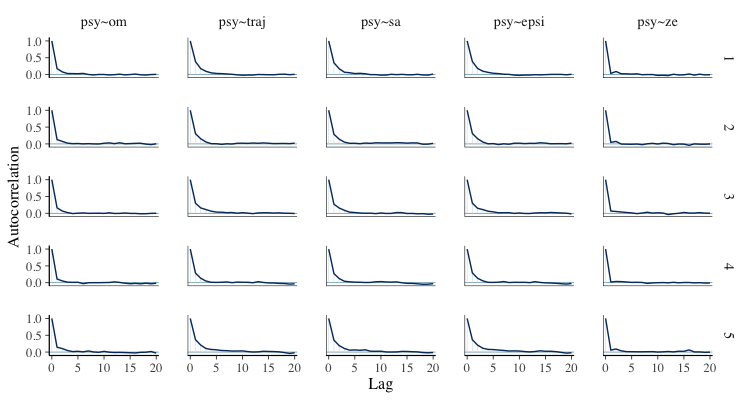


X axis shows the distance between current and past iterations, Y axis represents the correlation between those two iterations. Well-behaving chains are expected to fluctuate constantly (see the spread in the trace plots above) and thus show low autocorrelation with distant iterations, as is the case here. om: volatility $\omega$, traj: mean belief $\mu$, sa: uncertainty $\sigma$, epsi: Prediction error ε, ze: exploration ζ.

**Table S5**. Results of the structural equation model on latent trait psychopathy in high threat blocks

|  | Estimate | z | P |
| --- | --- | --- | --- |
| Factor loadings | | | |
| Interpersonal | .844 |  |  |
| Affective | .733 | 9.97 | <.001 |
| Lifestyle | .750 | 10.18 | <.001 |
| Antisocial | .575 | 7.69 | <.001 |
| Regression coefficients | | | |
| Volatility $\omega$ | -.239 | -2.01 | .014 |
| Mean belief $\mu$ | .644 | 2.59 | .001 |
| Uncertainty $\sigma$ | .400 | 2.68 | <.001 |
| Prediction error $\varepsilon$ | .582 | 2.09 | .001 |
| Exploration readiness $\zeta$ | -.010 | -0.13 | .893 |

Note that the first indicator (in this case, interpersonal) is fixed to one by default to set the scale of the latent variable and thus its p-value cannot be estimated. Z: z-score, p: p-value.

**Table S6**. Results of the structural equation model on latent trait psychopathy in low threat blocks

|  | Estimate | z | p |
| --- | --- | --- | --- |
| Factor loadings | | | |
| Interpersonal | .858 |  |  |
| Affective | .730 | 10.35 | <.001 |
| Lifestyle | .772 | 10.90 | <.001 |
| Antisocial | .556 | 7.64 | <.001 |
| Regression coefficients | | | |
| Volatility $\omega$ | .096 | 0.71 | .476 |
| Mean belief $\mu$ | .272 | 1.04 | .296 |
| Uncertainty $\sigma$ | .063 | 0.31 | .754 |
| Prediction error $\varepsilon$ | -.003 | -0.01 | .991 |
| Exploration readiness $\zeta$ | -.082 | 1.05 | .294 |

Note that the first indicator (in this case, interpersonal) is fixed to one by default to set the scale of the latent variable and thus its p-value cannot be estimated. Z: z-score, p: p-value.

**Table S7**. Results of the structural equation model on trait aggression in high threat blocks including participants with 50% missed trials (n=198)

|  | Estimate | z | p |
| --- | --- | --- | --- |
| Factor loadings | | | |
| Physical aggression | .677 |  |  |
| Verbal aggression | .689 | 7.90 | <.001 |
| Hostility | .644 | 7.50 | <.001 |
| Anger | .802 | 8.53 | <.001 |
| Regression coefficients | | | |
| Volatility $\omega$ | -.188 | -1.98 | .047 |
| Mean belief $\mu$ | .410 | 2.64 | .008 |
| Uncertainty $\sigma$ | .265 | 2.59 | .010 |
| Prediction error $\varepsilon$ | .373 | 2.29 | .022 |
| Exploration readiness $\zeta$ | .027 | 0.48 | .627 |

Note that the first indicator (in this case, physical aggression) is fixed to one by default to set the scale of the latent variable and thus its p-value cannot be estimated. Z: z-score, p: p-value.

**Table S8**. Results of the structural equation model on psychopathic traits in high threat blocks including participants with 50% missed trials (n=198)

|  | Estimate | z | p |
| --- | --- | --- | --- |
| Factor loadings | | | |
| Interpersonal | .851 |  |  |
| Affective | .748 | 10.67 | <.001 |
| Lifestyle | .764 | 10.87 | <.001 |
| Antisocial | .575 | 7.96 | <.001 |
| Regression coefficients | | | |
| Volatility $\omega$ | -.168 | -1.47 | .142 |
| Mean belief $\mu$ | .492 | 2.65 | .008 |
| Uncertainty $\sigma$ | .267 | 2.19 | .029 |
| Prediction error $\varepsilon$ | .362 | 1.85 | .064 |
| Exploration readiness $\zeta$ | -.003 | -0.05 | .959 |

Note that the first indicator (in this case, interpersonal) is fixed to one by default to set the scale of the latent variable and thus its p-value cannot be estimated. Z: z-score, p: p-value.

**Table S9**. Results of the structural equation model on latent trait aggression in high threat blocks including only women (n=124)

|  | Estimate | z | p |
| --- | --- | --- | --- |
| Factor loadings | | | |
| Physical aggression | .714 |  |  |
| Verbal aggression | .676 | 6.75 | <.001 |
| Hostility | .553 | 5.58 | <.001 |
| Anger | .871 | 7.73 | <.001 |
| Regression coefficients | | | |
| Volatility $\omega$ | -.170 | -1.81 | .070 |
| Mean belief $\mu$ | .707 | 3.56 | <.001 |
| Uncertainty $\sigma$ | .426 | 3.59 | <.001 |
| Prediction error $\varepsilon$ | .763 | 3.55 | <.001 |
| Exploration readiness $\zeta$ | -.036 | -0.65 | .627 |

Note that the first indicator (in this case, physical aggression) is fixed to one by default to set the scale of the latent variable and thus its p-value cannot be estimated. Z: z-score, p: p-value.

**Table S10**. Results of the structural equation model on latent trait aggression in high threat blocks including only men (n=64)

|  | Estimate | z | p |
| --- | --- | --- | --- |
| Factor loadings | | | |
| Physical aggression | .710 |  |  |
| Verbal aggression | .677 | 4.71 | <.001 |
| Hostility | .761 | 5.18 | <.001 |
| Anger | .783 | 5.27 | <.001 |
| Regression coefficients | | | |
| Volatility $\omega$ | -.370 | -1.63 | .101 |
| Mean belief $\mu$ | .441 | 0.86 | .387 |
| Uncertainty $\sigma$ | .377 | 1.07 | .283 |
| Prediction error $\varepsilon$ | .515 | 0.78 | .434 |
| Exploration readiness $\zeta$ | .119 | 0.81 | .414 |

Note that the first indicator (in this case, physical aggression) is fixed to one by default to set the scale of the latent variable and thus its p-value cannot be estimated. Z: z-score, p: p-value.

**Table S11**. Results of the structural equation model on latent trait psychopathy in high threat blocks including only women (n=124)

|  | Estimate | z | p |
| --- | --- | --- | --- |
| Factor loadings | | | |
| Interpersonal | .796 |  |  |
| Affective | .786 | 8.42 | <.001 |
| Lifestyle | .776 | 8.33 | <.001 |
| Antisocial | .572 | 6.09 | <.001 |
| Regression coefficients | | | |
| Volatility $\omega$ | -.138 | -1.17 | .240 |
| Mean belief $\mu$ | .706 | 2.92 | .003 |
| Uncertainty $\sigma$ | .444 | 3.08 | .002 |
| Prediction error $\varepsilon$ | .720 | 2.76 | .006 |
| Exploration readiness $\zeta$ | -.049 | -0.71 | .476 |

Note that the first indicator (in this case, interpersonal) is fixed to one by default to set the scale of the latent variable and thus its p-value cannot be estimated. Z: z-score, p: p-value.

**Table S12**. Results of the structural equation model on latent trait psychopathy in high threat blocks including only men (n=64)

|  | Estimate | z | p |
| --- | --- | --- | --- |
| Factor loadings | | | |
| Interpersonal | .907 |  |  |
| Affective | .617 | 4.77 | <.001 |
| Lifestyle | .655 | 5.06 | <.001 |
| Antisocial | .628 | 4.85 | <.001 |
| Regression coefficients | | | |
| Volatility $\omega$ | -.458 | -1.71 | .087 |
| Mean belief $\mu$ | -.003 | -.01 | .996 |
| Uncertainty $\sigma$ | .188 | .448 | .654 |
| Prediction error $\varepsilon$ | -.252 | 1.01 | .751 |
| Exploration readiness $\zeta$ | .179 | -0.05 | .309 |

Note that the first indicator (in this case, interpersonal) is fixed to one by default to set the scale of the latent variable and thus its p-value cannot be estimated. Z: z-score, p: p-value.

**Power analyses**

In addition to the excellent fit and converging results obtained using both frequentist and Bayesian approaches, we ran post hoc power analyses for the structural equation models linking latent aggressiveness and psychopathy factors with learning-related parameters. We used the semPower web application (Jobst et al., 2021) with the number of manifest variables, the degrees of freedom, and the obtained Goodness of Fit Index as input. With a threshold of p<.05, a sample size of n=188 confers about 92% (aggression model) and 98% (psychopathy model) statistical power to falsify the models in the event they were wrong. These estimations should however be taken with caution due to the known unreliability of post hoc power analyses (Zhang et al., 2019).

Jobst LJ, Bader M, Moshagen M. 2021. A tutorial on assessing statistical power and determining sample size for structural equation models. *Psychol Methods*. doi:10.1037/met0000423

Zhang Y, Hedo R, Rivera A, Rull R, Richardson S, Tu XM. 2019. Post hoc power analysis: is it an informative and meaningful analysis? *Gen Psychiatry* **32**:e100069. doi:10.1136/gpsych-2019-100069

**Figure S12**. Correlations between subscales of the aggressiveness and psychopathy questionnaires


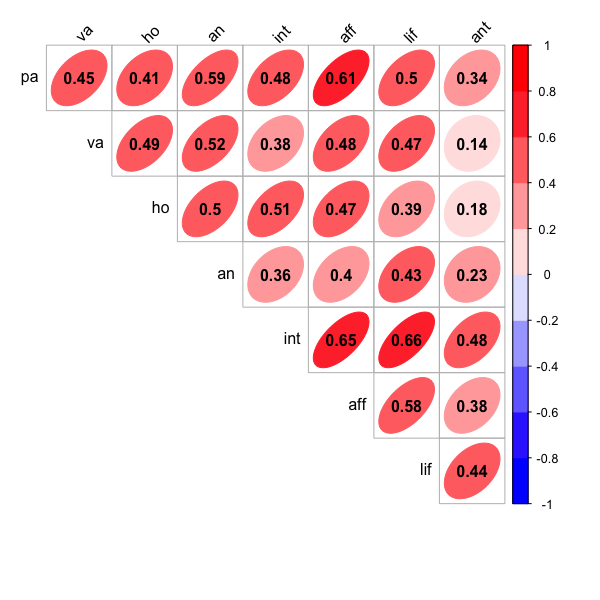


pa: physical aggression; va: verbal aggression; ho: hostility; an: anger; int: interpersonal manipulation; aff: callous affect; lif: erratic lifestyle; ant: antisocial/criminal tendencies. Values are Pearson correlation coefficients; more intense hues and elongated ellipses depict larger values. All correlations were significant after False Discovery Rate correction for multiple comparisons (q<.05). n=256.
